# Resistance to chemotherapy in advanced ovarian cancer: mechanisms and current strategies

**DOI:** 10.1038/sj.bjc.6601497

**Published:** 2003-12-17

**Authors:** P A Vasey

**Affiliations:** 1 Cancer Research UK, Department of Medical Oncology, Beatson Oncology Centre, Western Infirmary, Glasgow G11 6NT, UK

**Keywords:** ovarian cancer, drug resistance, chemotherapy, taxanes

## Abstract

Although treatment for advanced epithelial ovarian cancer has improved over recent years with the introduction of taxane–platinum chemotherapy, the majority of patients will relapse, and in most the disease remains incurable. A thorough understanding of drug resistance mechanisms is needed, as this remains the largest obstacle in treating patients with recurrent disease. Multidrug resistance proteins, mismatch repair processes and alterations in the p53 pathway are examples of properties within tumour cells that may lead to drug resistance. Novel agents designed to circumvent these mechanisms (e.g. PSC 833, ONYX-015 and ADP53) are currently being investigated for ovarian cancer patients. Further improvements may result from the optimisation of existing first-line regimens with more creative schedules, perhaps involving sequential or intraperitoneal administration of existing drugs, and the incorporation of newer noncross-resistant drugs.

Treatment for epithelial ovarian cancer has improved over the past 20 years with the introduction of the platinums and, more recently, taxane-based chemotherapy. The results of randomised-controlled trials have established paclitaxel in combination with a platinum agent as a standard initial chemotherapy for advanced ovarian cancer patients (McGuire *et al*, 1996; Piccart *et al*, 2000; du Bois *et al*, 2003; Ozols *et al*, 2003). Recent studies have suggested that docetaxel–carboplatin is as effective as paclitaxel–carboplatin chemotherapy (Vasey, 2001,^2002^).

Despite these treatment advances, most patients will relapse after achieving complete clinical response, and in the majority of these patients the disease is incurable (Lister-Sharp *et al*, 2000). As a result, the treatment of recurrent disease is an important aspect in the overall management of patients with epithelial ovarian cancer. A thorough understanding of drug resistance mechanisms is needed, as this remains the largest obstacle in treating patients with recurrent disease. Alterations to current taxane–platinum therapy such as the incorporation of newer noncross-resistant drugs, sequential therapy and intraperitoneal (i.p.) administration may help improve survival duration. This review examines some of the major mechanisms of resistance of tumours to anticancer drugs with particular reference to advanced epithelial ovarian cancer, and the means by which both clinical and laboratory researchers are seeking to circumvent these mechanisms in patients.

## CELLULAR MECHANISMS OF DRUG RESISTANCE

A wide range of metabolic or structural properties within tumour cells may lead to drug resistance. The identified mechanisms (Goldenberg, 1998) include:

- decreased drug uptake,
- increased drug efflux,
- increased repair of DNA damage induced by chemotherapy and
- reduced ability to undergo apoptosis.

In addition, because cancer cells are heterogeneous, more than one mechanism of drug resistance may be present in any particular case.

As shown in Table 1

**Table 1:** Mechanisms of *in vitro* drug resistance

**Taxanes**	**Platinum agents**
↑ Export from cells by P-glycoprotein	↓ Net uptake into cells
Mutations of tubulin	↓ Mismatch repair
↓ Apoptosis	↓ Apoptosis
Alterations in cell signalling (e.g. raf-1/bcl-2)	Alterations in activation and DNA repair
	Mutations in p53

, a number of specific factors have been identified as causes of taxane and/or platinum resistance *in vitro*, although the clinical relevance of the majority of these requires clarification (Kaye, 2000). Translational studies involving the prospective collection of tumour samples from the same patient before treatment and on clinical relapse are likely to be integral in ascertaining the clinical relevance of resistance. Circulating blood has been shown to contain free tumour DNA and tumour cells in most cancer patients (Leon *et al*, 1997). Sequential sampling of blood for molecular changes in blood tumour DNA may be a convenient means of monitoring the *in vivo* development of tumour resistance mechanisms.

### Classical multidrug resistance

Many drugs are substrates for membrane-based proteins (from the ATP-binding cassette family) that actively pump drugs out of cells. The expression of these proteins can cause multidrug resistance (MDR) towards numerous anticancer drugs, including the taxanes (Goldenberg, 1998). Two important MDR-associated proteins are P-glycoprotein and MDR-associated protein (MRP). One study investigated the role of MDR markers in predicting chemotherapy response in ovarian cancer patients by analysing samples taken from 58 patients at initial surgery (Yokoyama *et al*, 1999). The 5-year disease-free survival rate was 26.0% for patients with MRP-positive tumours and 72.5% for those with MRP-negative tumours. The prognosis for patients with MRP-positive tumours was significantly poorer (*P*<0.05), which suggests that MRP is a predictor of response to standard chemotherapy in ovarian cancer (Yokoyama *et al*, 1999).

Valspodar (PSC 833) is an MDR modulator — designed to reverse drug resistance-mediated through P-glycoprotein — that is currently undergoing clinical investigation. In a Phase I/II study, 59 patients with recurrent ovarian cancer who had failed prior platinum- and anthracycline-based chemotherapy were treated with valspodar (2, 4 or 10 mg kg^−1^ day^−1^) over 3 days, followed by doxorubicin (20–50 mg m^−2^) and cisplatin (50 mg m^−2^) on day 3 (Baekelandt *et al*, 2001). The addition of valspodar did not worsen cisplatin-related toxicity, and, of the 33 patients treated at the maximum-tolerated dose, one patient had a complete response (3%) and four (12%) had a partial response. In another study, 60 patients with refractory ovarian cancer were treated with valspodar (5 mg kg^−1^) four times daily for 12 doses and paclitaxel (70 mg m^−2^) on day 2, 2 h after the fifth or sixth dose of valspodar (Fracasso *et al*, 2001). The combination had limited activity (median progression-free survival (PFS) 1.5 months), although the authors suggested that patients with paclitaxel- and platinum-resistant tumours may not be an ideal trial population. It is hypothesised that initial treatment with valspodar in combination with taxane–platinum chemotherapy may suppress the emergence of resistant tumour cells. The first results of a large, multicentre, randomised trial of carboplatin AUC 6 plus paclitaxel 175 mg m^−2^ *vs* carboplatin AUC 6 plus paclitaxel 80 mg m^−2^ plus PSC 833 on days 0–3 of each cycle were presented at ASCO in 2002 (Joly *et al*, 2002). The dose of paclitaxel was reduced due to the increased toxicity observed in the previous studies when combined with PSC 833. Despite this, patients receiving chemotherapy plus PSC 833 demonstrated more myelotoxicity and emesis, in addition to ataxia — a toxicity largely unexplained, but again seen in Phase II studies. The chemotherapy-only arm demonstrated more neuropathy. However, there were no significant differences in the median time to progression (13.2 *vs* 13.5 months, HR.960). The overall survival was too premature, but this analysis suggests that MDR modulation in this manner may be of limited clinical relevance in ovarian cancer. It has been suggested that multiple mechanisms may need to be targeted to have any clinically important impact on outcome.

### Mismatch repair

Mismatch repair (MMR) proteins such as hMSH2 and hMLH1 recognise and repair damaged or mismatched DNA. Defects in the MMR pathway are an example of molecular events that may be associated with ovarian cancer resistance. Experimental data indicate that deficiencies in hMLH1 result in ‘replicative bypass’ following exposure to cytotoxic agents, by which DNA damage is not recognised and tumour cells continue to divide (Brown *et al*, 1997). The clinical relevance of MMR is currently being assessed via blood tumour DNA in a large-scale Scottish Gynaecological Trials Group study (SCOTROC 1). Blood samples from nearly 1000 patients (all prior to chemotherapy and as they relapse) are being collected and will be analysed for the presence of microsatellite instability (a DNA change indicative of defects in MMR). Approximately 70% of the first 150 patients analysed have abnormalities consistent with the presence of tumour DNA and 28% have microsatellite instability (Kaye, 2001). The analysis of samples collected after relapse is likely to be of key relevance in gauging the clinical importance of MMR in ovarian cancer resistance.

### p53 protein

The p53 protein is involved in controlling the progression of cells through the cell cycle and in mediating cellular responses to DNA damage through modulation of cell cycle regulation, DNA repair and activation of pathways leading to apoptosis (Benchimol and Minden, 1998). Mutations in the p53 gene are associated with a lack of response to high-dose cisplatin therapy in ovarian cancer patients (Righetti *et al*, 1996). Interestingly, preclinical investigations show that p53 mutations and the acquisition of cisplatin resistance are associated with increased sensitivity to taxanes in ovarian carcinoma cells (Cassinelli *et al*, 2001). The efficacy of the taxanes against mutant p53 ovarian cancer has been demonstrated in the clinical setting (Lavarino *et al*, 2000). Of 48 previously untreated patients with advanced disease, 34 (71%) responded to paclitaxel-based chemotherapy. Among the patients with mutant p53, 86% responded to therapy while only 47% of patients with wild-type p53 responded to the same therapy. Although other factors may be confounding, it may be possible to harness this increased sensitivity by targeting patients with platinum-resistant, p53-negative tumours for taxane therapy. Further studies are required in this area.

Genetic therapies are being explored as alternative approaches to circumventing p53-induced resistance in ovarian cancer (Wolf *et al*, 2000; Kigawa *et al*, 2001; Vasey *et al*, 2002). One such approach is the use of the adenovirus ONYX-015, attenuated so that it replicates in cells with absent or nonfunctional p53. This leads to virus spread and subsequent cytolysis of tumour cell populations. In a Phase I trial, 16 patients with recurrent ovarian cancer received i.p. ONYX-015. The maximum-tolerated dose was not reached at 1 × 10^11^ plaque-forming units; at this dose level patients selected for nonbulky i.p. disease did not experience significant dose-limiting toxicities. The evidence of virus replication was seen in the peritoneal fluid in one patient for up to 17 days (Vasey *et al*, 2002); this study therefore described the first clinical experience of any replication-competent/selective virus in cancer patients. Wolf *et al* (2000) have investigated a complementary approach in which wild-type p53 is reintroduced into tumour cells using adenovirus ADP53 in an attempt to restore chemosensitivity and promote apoptosis. Preliminary results from the Phase I study in 14 evaluable patients demonstrate that i.p. ADP53 is feasible and well tolerated (Wolf *et al*, 2000). A large-scale, randomised trial is underway to assess the potential of this approach in combination with platinum-based chemotherapy.

## STRATEGIES FOR MODULATING DRUG RESISTANCE

### Combination therapy with noncross-resistant agents

When ovarian cancer recurs, the goal of therapy shifts from cure to palliation. The treatment of recurrent ovarian cancer involves the use of second- or third-line drugs chosen for their lack of crossresistance with the primary agents employed. In contrast to the situation in previously untreated patients for whom prospective randomised trials have established the current taxane–platinum standards, there have been few randomised trials in patients with recurrent disease that demonstrate a survival advantage for one particular drug or regimen (Ozols, 2002). However, 2003 saw the results of the first adequately powered clinical trial in recurrent disease that demonstrated a survival advantage for combination chemotherapy over single-agent platinum (Parmar *et al*, 2003). Perhaps, more significant was the fact that there were no disadvantageous consequences (significant toxicity, quality of life) from the paclitaxel−carboplatin combination over platinum monotherapy in relapsed disease.

A series of single agents has been shown to have clinical activity in recurrent ovarian cancer; the most recent examples of these include topotecan, irinotecan (CPT-11), liposomal doxorubicin, gemcitabine and vinorelbine (Takeuchi *et al*, 1991; Burger *et al*, 1999; Ozols, 2002). Most notably, it has also been demonstrated that retreatment with either a platinum or a taxane is associated with significant clinical activity in patients with recurrent disease (Gore *et al*, 1990; Markman *et al*, 1991; Thigpen *et al*, 1994; Abu-Rustum *et al*, 1997). For example, single-agent docetaxel is an active second-line agent in patients refractory to both paclitaxel- and platinum-based regimens (Kavanagh *et al*, 1996; Katsumata *et al*, 2000; Verschraegen *et al*, 2000), which suggests that resistance to a specific agent does not necessarily mean that the tumour will not respond to other agents in the same class.

The treatment-free interval (TFI), defined as the period of time between the end of first-line chemotherapy and the start of second-line therapy, may assist in deciding on a second-line therapy. Markman *et al* (1991) have shown that a TFI of between 6 and 24 months predicts for a response rate of 30% to second-line platinum, while a TFI >24 months predicts for a response rate of approximately 60%. It has since been suggested that it may be possible to increase the platinum-free interval by using an alternative agent first, thereby saving platinum for later (Bookman, 1999; Cannistra, 2002). The taxanes, topotecan and liposomal doxorubicin have potential as alternative agents in this setting. In support of this, a subset of platinum-resistant patients were converted to a platinum-sensitive state through the interval use of paclitaxel in a small trial (*n*=33), presumably by allowing the emergence of platinum-sensitive tumour cells (Kavanagh *et al*, 1995). Likewise, a similar study demonstrated a response rate of 26% to platinum rechallenge in patients considered platinum resistant by treatment with nonplatinum agents in the intervening period (Spriggs *et al*, 2002). Laboratory data have hinted that the underlying mechanism for this may be the downregulation with time — in the absence of platinum — of excision repair enzymes, known to be overexpressed in resistant disease (Dabholkar *et al*, 1992; Yu *et al*, 2000). On the basis of these observations, the concept of prolonging the platinum-free interval is a strategy that some physicians have already adopted in clinical practice (Cannistra, 2002). However, such a strategy requires randomised trial data, and two studies are addressing this — Gynaecologic Oncology Group (GOG) trial 202 (this is primarily directed at investigating the role of secondary cytoreduction) and the planned Optimum Sequence of Chemotherapy At Relapse, OSCAR trial in the UK.

### Sequential therapy

Sequential therapy is an approach designed to avoid the development of drug resistance. One agent is used initially and then treatment is switched in order to — in principle — eliminate cells that may have developed resistance to continued use of the initial therapy (Kaye, 2000). Potential advantages are that full doses of the most potent agent can be administered initially and differential sensitivities of tumour cells to certain agents can be exploited. Such regimens may offer improved tolerability and reduced risk of negative interactions between agents. This approach has proved successful in breast cancer for a number of regimens, including sequential docetaxel followed by doxorubicin and cyclophosphamide (Khayat *et al*, 2001), and sequential docetaxel and 5-fluorouracil–epirubicin–cyclophosphamide (Spielmann *et al*, 2002).

The numerous active drug classes in ovarian cancer mean that many sequential chemotherapy regimens are possible. Although there is a current lack of completed trials using a truly sequential approach, one GOG trial 132 does hint at the value of sequential taxane therapy in ovarian cancer (Muggia *et al*, 2000). This three-arm study compared paclitaxel 135 mg m^−2^ over 24 h plus cisplatin 75 mg m^−2^ with cisplatin 100 mg m^−2^ or paclitaxel 200 mg m^−2^ over 24 h alone. The results showed no significant difference in overall survival among treatment arms, although cisplatin alone or in combination yielded significantly higher tumour response rates than paclitaxel monotherapy. It should be noted, however, that early treatment crossover between groups was frequent in this trial, and that paclitaxel therapy was frequently initiated after cisplatin-only therapy. This use of nonprotocol therapy was attributable principally to the presence of persistent disease as determined either clinically or by reassessment surgery. The similarity of results from the initial cisplatin (and crossover paclitaxel) and combination arms suggests that sequential therapy may be an effective approach. However, the trial was not designed to determine the effect of sequential therapy and further study is therefore needed to clarify this issue.

Feasibility trials for sequential therapy in ovarian cancer are currently underway in Europe and the US. The Scottish Gynaecologic Cancer Trial Group SCOTROC-2 study programme is currently recruiting patients in the UK and Europe (Kaye, 2001). This series of trials is designed to assess four cycles of single-agent carboplatin followed by a combination of docetaxel (or paclitaxel) with CPT-11 or gemcitabine as first-line therapy in patients with advanced ovarian cancer. First results of SCOTROC 2A, in which gemcitabine was the agent being evaluated, have demonstrated that this approach is feasible and highly active (Rustin *et al*, 2002; Vasey *et al*, 2003). In an ongoing, randomised, German Arbeitsgemeinschaft Gynaekologische Onkologie (AGO) trial, patients receive paclitaxel–carboplatin chemotherapy followed by sequential topotecan. A feasibility trial for this approach was successfully completed (Sadoze *et al*, 1999), and the results from the randomised study were presented at ASCO 2003 (Pfisterer *et al*, 2003). Although feasible with regard to deliverability and toxicity, early data do not demonstrate an improvement in PFS for this approach. However, the primary end point of the study was overall survival, and further follow-up is still required. Finally, another alternative is to utilise the administration of sequential or alternating doublets for as many as eight cycles of treatment — this potentially retains the concept of preventing drug resistance while introducing new agents in a less toxic way. A five-arm, prospective, randomised trial is in progress by the Southwest Oncology Group (SWOG), the Gynecologic Oncology Group (GOG), the European Organisation for Research and Treatment of Cancer (EORTC), the Medical Research Council (MRC) and the National Cancer Institute of Canada (NCIC), with an accrual goal of approximately 5000 patients. This study — GOG 182 (ICON 5 in Europe) — is comparing four cycles of gemcitabine–carboplatin followed by four cycles of paclitaxel–carboplatin *vs* four cycles of liposomal doxorubicin/paclitaxel–carboplatin followed by four cycles of paclitaxel–carboplatin *vs* four cycles of topotecan–carboplatin followed by four cycles of paclitaxel–carboplatin *vs* eight cycles of gemcitabine–paclitaxel–carboplatin *vs* eight cycles of paclitaxel–carboplatin (control arm).

### Dose-intense chemotherapy

Increased exposure to cytotoxic drugs has been used as a means of circumventing drug resistance and potentially increasing the response rates and survival times. A variety of randomised clinical trials have been carried out to assess dose intensification of platinum therapy, two of which showed a significant improvement in survival with high-dose treatment (see review by Vasey and Kaye, 1997).

The earliest of these studies (Ngan *et al*, 1989) was carried out in 50 assessable patients with debulked advanced epithelial ovarian cancer, and showed 3-year actuarial survival rates of 60 and 30% for high-dose (120 mg m^−2^) and low-dose (60 mg m^−2^) cisplatin, respectively (both given every 3–4 weeks in combination with cyclophosphamide 600 mg m^−2^). Notwithstanding this impressive difference between groups, some doubt has been attached to the results of this study because of the small size of the patient cohort studied and because baseline disease characteristics were not fully characterised. A later and larger study carried out by the Scottish Gynaecologic Cancer Trials Group (159 patients) compared cisplatin 50 with 100 mg m^−2^, with either dose given every 3 weeks with cyclophosphamide 750 mg m^−2^, and showed respective overall survival rates of 32.4 and 26.6% (overall relative death rate=0.68; *P*=0.043) over 4 years after the start of the trial (Kaye *et al*, 1996).

These results — while interesting — should be viewed in light of other studies that have shown no significant difference between high- and low-dose approaches (Vasey and Kaye, 1997). However, many of these studies only aim to deliver up to two-fold increases in platinum dose intensity; higher doses are not achievable in the clinical setting without bone marrow or stem cell support. In addition, substantial toxicity — particularly bone marrow and neurotoxicities — was evident with the higher-dose cisplatin treatment in both the above trials; to date, no large randomised studies evaluating a role for true high-dose chemotherapy have been performed. The conclusions from these data are that increasing dose intensity two-fold has no impact on outcome, and high-dose approaches are currently investigational and should be reserved for properly designed clinical trials.

### Intraperitoneal therapy

For over 20 years, investigators have explored the role of chemotherapy administered directly into the peritoneal cavity for patients with ovarian cancer (Jones *et al*, 1978). In principle, the strategy allows tumour cells to be exposed to higher doses of chemotherapy than would otherwise be possible with systemic therapy. Ovarian cancer remains confined to the peritoneal cavity for much of its natural history, making the disease an ideal candidate for such a drug delivery strategy. The first randomised trial exploring i.p. therapy as part of primary treatment in ovarian cancer incorporated intravenous (i.v.) cyclophosphamide (600 mg m^−2^) and i.v. or i.p. cisplatin (both at a dose of 100 mg m^−2^) in >600 women with stage III ovarian cancer (Alberts *et al*, 1996). A significant survival advantage in patients treated with i.p. cisplatin compared with those in the i.v. cisplatin arm was observed (49 *vs* 41 months, respectively; *P*=0.02). There was significantly reduced neutropenia and tinnitus, and an expected increase in abdominal comfort observed in the i.p. arm. There are, nevertheless, problems with the correct interpretation of this study. Accrual was extended to include more patients with residuum ⩽0.5 cm, the investigators rationalising that this group would benefit most from i.p. chemotherapy. Counterintuitively, there turned out to be no statistically significant survival benefit for this group. In addition, as the i.v. cisplatin–cytoxan combination has been shown to be inferior to cisplatin–paclitaxel in two randomised studies, it is therefore not considered to be an appropriate control arm with which to gauge any new therapy.

A subsequent randomised Phase III study has incorporated a taxane into the treatment regimen (Markman *et al*, 2001). The experimental arm comprised i.p. cisplatin (100 mg m^−2^) and i.v. paclitaxel (135 mg m^−2^), while the control arm consisted of i.v. cisplatin (75 mg m^−2^) and i.v. paclitaxel (135 mg m^−2^). The experimental treatment was associated with a statistically significant improvement in PFS (28 *vs* 22 months; *P*=0.01), but there was no significant improvement in overall survival. This trial suffers from a number of design flaws, not least of which is the fact that delivering two cycles of carboplatin at AUC 9 in addition to six cycles of cisplatin–paclitaxel adds a longer duration of chemotherapy and an increased cumulative dose of platinum, thus unbalancing the trial in favour of the research arm, irrespective of the mode of administration. In addition, the timing of salvage therapies is not known, and may have influenced the PFS end point if administered before progression (salvage therapies are also likely to have influenced the overall survival, and were not controlled). Furthermore, considerably greater toxicity was observed in the experimental i.p. arm (grade IV thrombocytopenia and grade III gastrointestinal).

Finally, the preliminary results of GOG trial 172 were presented at ASCO in 2002 (Armstrong *et al*, 2002). This study randomised 417 patients cytoreduced to ⩽1 cm residuum to receive either six cycles of i.v. cisplatin plus i.v. paclitaxel or six cycles of a 3-weekly regimen consisting of i.v. paclitaxel (day 1), i.p. paclitaxel (day 2) and i.p. paclitaxel (day 8). PFS was significantly longer in the i.p. arm (24 *vs* 19 months, *P*=0.029), although again there was significantly greater grade 3–4 toxicity — especially metabolic, neurological and gastrointestinal events and infections.

These results from three relatively large-scale clinical trials carried out to date are all therefore positive for the i.p. approach and — despite the criticisms outlined here — there may be an emerging benefit accruing from this approach. As opined in a recent editorial in the *Journal of Clinical Oncology*, ‘it is difficult to think of any other setting in oncology where the results of three positive trials have not led to widespread adoption of the superior therapy’ (Alberts *et al*, 2002). The prevailing belief against i.p. therapy may need to be overcome — and a less toxic regimen for i.p. chemotherapy designed — before this treatment becomes part of routine clinical practice.

## DRUGS IN THE PIPELINE

As shown in Table 2

**Table 2:** Novel anticancer agents in development

**Drug class**	**Agent**
Signal transduction inhibitors	Monoclonal antibodies (e.g. anti-Her2/neu [trastuzumab])
	EGFR tyrosine kinase inhibitors (e.g. ZD1839, OSI 774)
Angiogenesis inhibitors	VEGFR tyrosine kinase inhibitors (e.g. SU6668)
	Thalidomide
Genetic therapy	Circumvention of p53 resistance (e.g. ADP53, ONYX-015)
	E1A gene lipid complex

EGFR=epidermal growth factor receptor; VEGFR=vascular endothelial growth factor receptor.

, there are numerous novel agents in development for the treatment of ovarian cancer, including those designed to circumvent drug resistance mechanisms, signal transduction inhibitors and angiogenesis inhibitors. Of particular interest are the EGFR tyrosine kinase inhibitors. A number of tumour types, including epithelial ovarian cancer, have a strong association between levels of EGFR and decreased survival (Nicholson *et al*, 2001). Aberrant EGFR activation is an important factor in tumorigenesis and an essential driving force for aggressive growth behaviour (Herbst and Shin, 2002). The binding of growth factors (e.g. EGF) activates cell surface receptors, which causes dimerisation (there are coreceptors, for example, HER2/neu). Subsequent receptor autophosphorylation on tyrosine residues occurs and these serve as docking sites for a number of signal transducers and adaptor molecules. The end result is a reaction cascade that leads to changes in proliferation, adhesion — which are responsible for uncontrolled cellular growth — and enhanced neovascularisation. Tyrosine kinase inhibitors such as OSI 774 (Tarceva®) are small molecules that compete internally for the ATP-binding pocket catalytic domain of the receptor tyrosine kinase. These and other agents in this class can be shown to be active as monotherapy in refractory ovarian cancer (Finkler *et al*, 2001) and may have synergism with platinum and taxanes. Future trial designs utilising these and other novel biologic agents will need to be careful to exploit their potential fully.

## CONCLUSIONS

Further advances in the treatment of women suffering from ovarian cancer will likely result from an improved understanding of clinical drug resistance, optimal use of existing drugs (in particular, the platinums and the taxanes) in new regimens (including possible developments in sequential or i.p. chemotherapy) and the integration of novel agents into current combinations. Multidrug resistance proteins, MMR processes and alterations in the p53 pathway are examples of properties within tumour cells that may lead to drug resistance. Novel agents designed to circumvent these mechanisms (valspodar, ONYX-015 and ADP53) are currently being investigated for ovarian cancer patients.
